# Deep learning-based segmentation and classification of leaf images for detection of tomato plant disease

**DOI:** 10.3389/fpls.2022.1031748

**Published:** 2022-10-07

**Authors:** Muhammad Shoaib, Tariq Hussain, Babar Shah, Ihsan Ullah, Sayyed Mudassar Shah, Farman Ali, Sang Hyun Park

**Affiliations:** ^1^ Department of Computer Science, CECOS University of Information Technology (IT) and Emerging Sciences, Peshawar, Pakistan; ^2^ High Performance Computing and Networking Institute, National Research Council (ICAR-CNR), Naples, Italy; ^3^ College of Technological Innovation, Zayed University, Dubai, United Arab Emirates; ^4^ Department of Robotics and Mechatronics Engineering, Daegu Gyeonbuk Institute of Science and Engineering (DGIST), Daegu, South Korea; ^5^ Institute of Computer Science & Information Technology, The University of Agriculture Peshawar, Peshawar, Pakistan; ^6^ Department of Software, Sejong University, Seoul, South Korea

**Keywords:** plant disease detection, deep learning, U-Net CNN, inception-net, object detection and recognition

## Abstract

Plants contribute significantly to the global food supply. Various Plant diseases can result in production losses, which can be avoided by maintaining vigilance. However, manually monitoring plant diseases by agriculture experts and botanists is time-consuming, challenging and error-prone. To reduce the risk of disease severity, machine vision technology (i.e., artificial intelligence) can play a significant role. In the alternative method, the severity of the disease can be diminished through computer technologies and the cooperation of humans. These methods can also eliminate the disadvantages of manual observation. In this work, we proposed a solution to detect tomato plant disease using a deep leaning-based system utilizing the plant leaves image data. We utilized an architecture for deep learning based on a recently developed convolutional neural network that is trained over 18,161 segmented and non-segmented tomato leaf images—using a supervised learning approach to detect and recognize various tomato diseases using the Inception Net model in the research work. For the detection and segmentation of disease-affected regions, two state-of-the-art semantic segmentation models, i.e., U-Net and Modified U-Net, are utilized in this work. The plant leaf pixels are binary and classified by the model as Region of Interest (ROI) and background. There is also an examination of the presentation of binary arrangement (healthy and diseased leaves), six-level classification (healthy and other ailing leaf groups), and ten-level classification (healthy and other types of ailing leaves) models. The Modified U-net segmentation model outperforms the simple U-net segmentation model by 98.66 percent, 98.5 IoU score, and 98.73 percent on the dice. InceptionNet1 achieves 99.95% accuracy for binary classification problems and 99.12% for classifying six segmented class images; InceptionNet outperformed the Modified U-net model to achieve higher accuracy. The experimental results of our proposed method for classifying plant diseases demonstrate that it outperforms the methods currently available in the literature.

## Introduction

We have been domesticating animals and cultivating crops for centuries. Agriculture enabled all of this to be possible. Food insecurity is the primary cause of plant infections (Chowdhury, et al., 2020; Global Network Against Food Crisis, 2022). It is also one of the reasons why humanity faces grave problems. One study indicates that plant diseases account for approximately 16 percent of global harvest yield losses. The global pest and disease losses for wheat and soybean are anticipated to be approximately 50 percent and 26 to 29 percent, respectivel (Prospects and Situation, 2022). The classifications of plant pathogens include fungi, fungus-like species, bacteria, viruses, virus-like organisms, nematodes, protozoa, algae, and parasitic plants. Artificial intelligence and machine vision have benefited numerous applications, including power forecasting from non-depletable assets (Khandakar et al., 2019; Touati et al., 2020) and biomedical uses (M. H. Chowdhury et al., 2019; Chowdhury et al., 2020). Artificial intelligence is beneficial. It has been utilized globally for the identification of lung-based diseases. In addition, this method has accumulated predictive applications for the virus (Chowdhury et al., 2021). Using these comparative trend-setting innovations, early-stage plant diseases can be identified. AI and computer vision are advantageous for the detection and analysis of plant infections. As physically inspecting plants and detecting diseases is a very laborious and tiring process, there is a chance of error. Consequently, the use of these techniques is very advantageous because they are not particularly taxing, they do not require a great deal of labour, and they reduce the likelihood of error. Sidharth et al. (Chouhan et al., 2018) utilized a distributed premise work organization (BRBFNN) with an accuracy of 83.07 percent. This network is used to improve bacterial searching to identify and organize plant diseases1. The convolutional neural network is a well-known neural organization that has been successfully applied to a variety of computer vision tasks (Lecun and Haffner, 1999). Different CNN structures have been utilized by analysts to classify and distinguish evidence of plant diseases. For instance, “Sunayana et al. compared various CNN structures for recognizing potato and mango leaf infection, with AlexNet achieving 98.33 percent accuracy and shallow CNN models achieving 90.85 percent accuracy (Arya and Singh, 2019)”. “Using the mean of the VGG16 model, Guan et al. predicted the disease severity of apple plants with a precision rate of 90.40 percent. They utilized a LeNet model (Wang, 2017; Arya and Singh, 2019) Jihen et al. employed a model known as LeNet. This model was used to identify healthy and diseased banana leaves with a 99.72 percent accuracy rate (Amara, et al., 2017).

Tomato is one of the most commonly consumed fruits on a daily basis. As tomatoes are utilized in condiments such as ketchup, sauce, and puree, their global utilization rate is high. It constitutes approximately fifteen percent (15%) of all vegetables and fruits, with an annual per capita consumption of twenty kilograms. An individual in Europe consumes approximately thirty-one (31) kilograms of tomatoes per year. In North America, this percentage is relatively high. A person consumes approximately forty-two (42) kilograms of tomatoes annually (Laranjeira et al., 2022). The high demand for tomatoes necessitates the development of early detection technologies for viruses, bacterial, and viral contaminations. Several studies have been conducted using technologies based on artificial intelligence. These technologies are used to increase tomato plants’ resistance to disease. “Manpreet et al. characterized seven tomato diseases with a 98.8 percent degree of accuracy (Gizem Irmak and Saygili, 2020)”. The Residual Network was utilized to classify and characterize these diseases. This residual network is built utilizing the CNN architecture. This network is generally known as ResNet. Rahman et al. (Rahman et al., 2019) projected a network with 99.25 percent accuracy. This network is utilized to determine how to distinguish bacterial spots, late blight, and segregation spots from tomato leaf images. Fuentes et al. (Fuentes et al., 2017) employed three distinct types of detectors. These detectors were used to differentiate ten diseases from images of tomato leaf. A convolutional neural network is one type of detector. This network is comprised of faster regions. The second detector is a network of convolutions. The third detector is a multi-box finder with a single shot (SSD). These indicators are coupled with a variety of deep component extractor variants. The Tomato Leaf Disease Detection (ToLeD) model, proposed by Agarwal et al. is CNN-based technology for classifying ten infections from images of tomato leaf with an accuracy of 91.2%. Durmus et al. (Durmus, et al., 2017) classified ten infections from images of tomato leaves with 95.5% accuracy using the Alex Net and Squeeze Net algorithms. Although infection grouping and identification of plant leaves are extensively studied in tomatoes, few studies include segmented leaf images from their specific environments. The function also occurred in other plant leaves; however, no studies have segmented images of leaves from their specific case. Since lighting conditions can drastically alter an image’s accuracy, improved segmentation techniques could help AI models focus on the area of interest rather than the setting.

U-net derives its moniker from its U-shaped network design. It is an architecture for cutting-edge image segmentation technology based on deep learning. U-net is designed to aid in the segmentation of biomedical images (Navab et al., 2015). In addition, unlike conventional CNN models, U-net includes convolutional layers for up-sampling or recombining feature maps into complete images. The experimental results of research articles (Rahman et al., 2020) demonstrated promising segmentation performance. The segmentation results are summarized using the cutting-edge U-Net model (Navab et al., 2015). In contrast, Inception Net is a modernized network. In addition, the Inception Net network is classification-based network used to predict the health condition of the crop using the tomato leaf data  (Louis, 2013). The main contribution of the proposed model can be summarized as:

1) Different U-net versions were explored to choose the optimum segmentation model by comparing the segmented model mask with the images of ground truth masks.
2) This study used three different classification methods: A comparison of different CNN architecture for classification tasks involving binary and multiclass classification of tomato diseases. Several experiments were carried out with various CNN architectures. (a) Binary classification of healthy and ill leaves on a scale of one to ten. (b) Two-level classification of healthy leaves and four levels of categorization for ill leaves (five levels total). (c) A ten-level categorization of healthy individuals, as well as nine illness categories. A twofold characterization of solid and infected leaves, a five-level order of sound, four unhealthy leaves, a ten-level grouping of solid, and nine disease classes.
3) The results achieved in this study outperform the most current state-of-the-art studies in this field in terms of accuracy and precision.

The remaining paper is organized as follows: The first section contains a detailed introduction, a review of literature, and the study’s motivation. Various kinds of pathogens that attacked plants are described in Section no. 2. Section 3 contains information about the study’s methodology and techniques, including a description of the dataset, pre-processing techniques, and experimental details. In section no. 4, the study’s findings are reported, including discussion in section no. 5 in Section 4, and then in the 6th section.

## Literature review

### Convolutional neural networks deep

Tan et al.  (Louis, 2013) introduced the Inception Net CNN model. We performed transfer learning for the detection of various tomato plant leaf diseases. The developer, Inception Net CNN model, ensured that the model is balance in all aspects, i.e., width, resolution, and depth. Moreover, the developers of Inception Net were the first to find the connection b/w all of the three dimensions, while other CNN ranging techniques use the single-dimensional ranking factor.

The writers used the MnasNet network (Sevilla et al., 2022) to create their baseline architecture, prioritizing model accuracy and FLOPA using a neural network architecture to search multi-objects. Next, they built the InceptionNet2, which was the same as MnasNet but with only one difference, i.e., more extensive than the Inception Net network. It happened because Inception Net’s FLOPS target is higher. Its crucial building block is the mobile reverse bottleneck MBConv (Sandler et al., 2018), including squeezing and excitation optimization (Hu et al., 2020). Finally, we employed a composite way, that composition method is based on InceptionNet1, which employs compound coefficients σ to scale. Using this scale, the neural network width, depth and dimensions can be detected. All of these three criteria are detected uniformly using the Equation below:


x ≥ 1, y ≥ 1, z ≥ 1                                                                                  (1)

We use a, b and c, as the following constant variables that can be recognized with the help of an efficient grid scan. The alpha value is the constant coefficient declared by a user, which adjusts the number of possessions utilized for scaling up the model. The values a, b, and c determine how these additional resources can be assigned to the neural network’s width, dimension, and depth. Here are some constants a, b, and c that may be discovered by quickly scanning the grid. As shown in the following Equation, the parameters for network width, depth, and resolution are defined by the variables a through c. The parameter may set the parameter for model scaling, represented by the parameter for model scaling.

It creates a family of Inception Net (1 to 2) by scaling up the reference point of the system and setting the constants as a, b, and c while scaling up the network reference point with various a, b, and c networks in **Table 1**. The accuracy of 97.1 percent achieved by InceptionNet1 on ImageNet is in the top five, even though on inference it is 6.1 times quicker and 8.4 times smaller than the finest existing ConvNets such as SENet (Hu et al., 2020) and Gpipe (Huang et al., 2019).

**Table 1:** Parameters of Inception Nerual Network.

Stage	Operator	Image dimesnion	No of channels	No of layers
1	Convolutional [3x3]	224x224	32	1
2	MobileConv 1, [3x3]	112x112	16	1
3	MobileConv 6, [3x3]	112x112	24	2
4	MobileConv 6, [3x3]	56x56	40	2
5	MobileConv 6, [3x3]	28x28	80	3
6	MobileConv 6, [5x5]	14x14	112	3
7	MobileConv 6, [5x5]	14x14	192	4
8	MobileConv 6, [3x3]	7x7	320	1
9	Convolitonal [1x1], Pooling and Fully Connected	7x7	1280	1

The InceptionNet1, InceptionNet3 and InceptionNet2 algorithms were utilized while constructing our design; A GAP layer was added to the network’s last layer to increase accuracy while also reducing overfitting. We added a thick layer Ensuing GAP, with a 1024x1024 resolution and a 25% loss. Followed by another Dense layer. A SoftMax layer is then applied to produce the likelihood estimate points for identifying leaf diseases of tomato, which is the final step to begin with Inception Net as a baseline; we scale it up in two steps using our compound scaling method:

The first step is to fix = one, assuming twice as many resources are available. The InceptionNet2 finest values for are, in particular, a,b,c at 1.2,1.1,1.15 respectively with a = 1.2 being the best overall.

Second, we fix the values of constants as a, b and c and utilize Equation (1) to scale up the baseline variety of network values to get InceptionNet2 through InceptionNet1.

In some instances, looking for the three variables near a big model, for example, can provide even better results. Still, the cost of the search becomes prohibitively expensive for larger models. The First step in addressing this issue is performing a single search on a tiny baseline network and scaling all other models in the second step with the same scaling coefficients as the small baseline network.

### Segmentation

**Figure 1:**
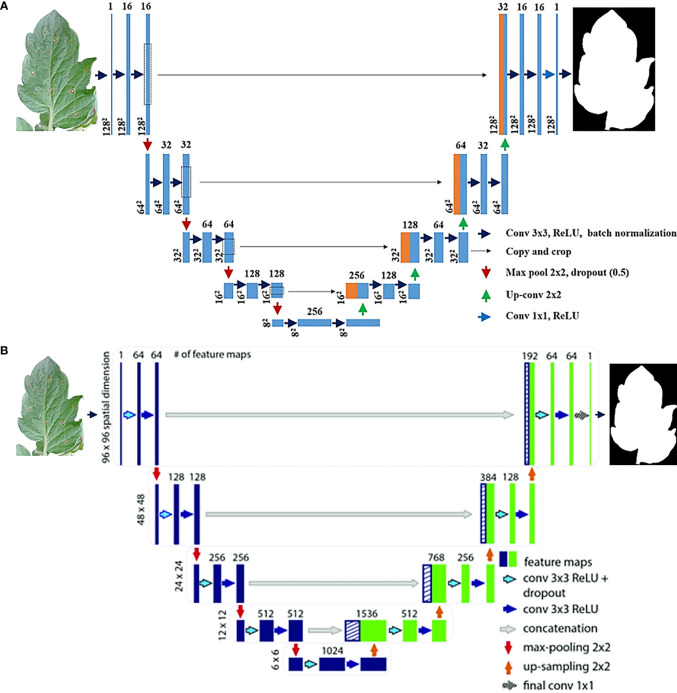
**(A)** Original Baseline U-Net Architecture, **(B)** Modified improved U-net Deep Neural Network Architecture Livne et al., 2019.

**Figure 2:**
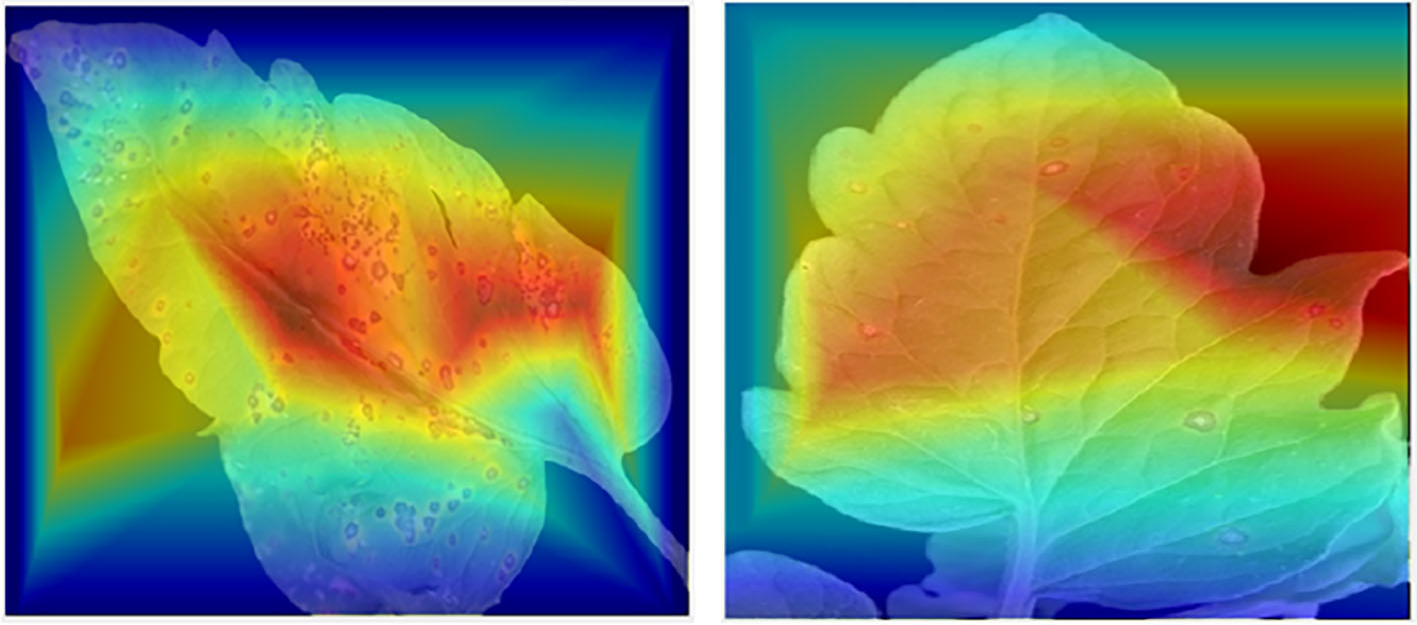
Sisualization of tomato leaf images using the Score-CAM tool, demonstrating affected regions where CNN classifier makes the majority of its decisions.

In U-net architecture, many segmentation designs may be built. This research compared two versions of the unique U-Net (Navab et al., 2015) and two distinct versions of the Modified U-Net (Navab, 2020) to see which version performed the best. You can see how the original U-Net design, the Improved U-Net design, and the Modified U-Net design are displayed in **Figures 1**, **2**. When considering the U-net, the first thing to bear in mind is that it comprises two pathways: one that expands while contracting and another that contracts while extending. An unpadded convolution (or convolution with padding) is performed many times along the contracting route. Each iteration consists of a ReLU followed by a pooling operation with stride 2 for down sampling. In the latter stages of the expanding path, the third convolution is followed by a ReLU. The up-sampled feature map is combined with the contracting path’s feature map, which is doubled, and two 3 x 3 convolutions, followed by a ReLU in the contracting approach. Every stage of the network takes into account 23 convolutional layers.

This research used a modified U-Net (Manjunath and Kwadiki, 2022) model, which includes some modest changes to its decoding component. U-Net refers to a route that provides for four encoding blocks and four decoding blocks, after which there is a second route that expands the first route by adding four encoding blocks and four decoding blocks. The decoding block of the new U-Net design uses three convolutional layers rather than two, which results in a substantial improvement in decoding block performance. Every block in every encoded picture has two 3x3 convolutional layers, and then the layers are repeated for each encoded image. During the up-sampling phase, the algorithm processes a more extensive set of images in the training set. Then, two three-by-three convolutional layers, one concatenation layer, and another three-by-three convolutional layer come into play. Convolutional layers also conduct batch normalization and ReLU activation. A 1 x 1 convolution is performed at each pixel on the SoftMax result from the previous layer. By allowing the final layer to differentiate between background and object pixels, this feature increases image quality. At the layer level of abstraction, this classification is carried out.

### Visualization techniques

CNNs are showing a greater interest in internal mechanics. Therefore, visualization approaches have been developed to aid in their understanding. Visualization methods help in the knowledge of CNN decision-making processes. Additionally, this makes the model more understandable to people, helping increase the faith in the findings of neural networks. Recently, “Score-CAM (Chen and Zhong, 2022) was employed in this investigation because of its good output, such as Smooth Grad (Smilkov et al., 2017), Grad-CAM (Selvaraju et al., 2020), Grad-CAM++ (Chattopadhay et al., 2018), and Score-CAM (Wang et al., 2020).” The weight for each activation map is based on the target class’s forward passing score, and the outcome is the product of weights and activation maps. After calculating the forward passing score for each activation map, Score-CAM removes the requirement for gradients. In **Figure 2**, the leaf areas were in control of CNN decision-making, as seen by the heat map. The statement above can assist consumers in understanding how the network makes decisions, which increases end-user confidence.

### Pathogens of tomato leaves

Septoria leaf spot, Early blight,target spot, and molds of leaf are only a few of the plant diseases caused by a fungus that exists. Fungi may infect plants in a change of ways *via* seeds and soil. The pathogenic fungus can spread across plants *via* animals, human contact, equipment, and soil contamination. An infection of the plant’s leaves by a fungal pathogen is the cause of initial blight tomato plants disease. All terms used to describe this condition are fruit rot, stem lesion, Collar rot and. When fighting early blight, cultural control, which includes fungicidal pesticides and good soil and nutrient management, is essential. Septoria leaf spot is caused by a fungus that grows on tomato plants and produces tomatines enzyme, which causes the breakdown of steroidal glycoalkaloids in the tomato plant to occur. Known as spot disease, it is a fungal disease that affects tomato plants and manifests itself as necrosis lesions with a color displayed as mild brown in the center. Defoliation occurs early in the course of progressive lesions (Pernezny et al., 2018; Abdulridha et al., 2020).

When the goal location is struck, it causes immediate harm to the fruit. This illness, called the fungal disease, develops upon moist leaves remain for a lengthy period. Bacteria is also a type plant pathogen. Bites, trimming, and cuts allow insects to penetrate plants. The availability, humidity, Temperature, nutrient meteorological conditions, ventilation, and soil conditions are crucial for bacterial development and plant harm. Bacterial spot is a disease caused by bacteria (Louws et al., 2001; Qiao et al., 2020). Plants can spread illness because of mold growth. Mold Plant causes the late blight in tomato and potato plants stems and leaf tips might have dark, irregular blemishes. The Tomato Yellow Leaf Cur (TYLC) virus causes illness in tomatoes. This virus has infected the plant and is transmitted by an insect. However, tomato plants bear damaged leaves and are divided into ten different classifications. In research 2, several groups of unhealthy and stable leaf photographs were categorized.

Some investigations show that plants of beans, peppers, eggplant and tobacco can also be harmed by virus. The current priority is to combat yellow leaf curl disease due to the illness’s extensive geographical range. Tomato Mosaic Virus is also a type pathogen which impacts the tomato plants (Ghanim et al., 1998; Ghanim and Czosnek, 2000; Choi et al., 2020; He et al., 2020). This virus is prevalent everywhere, affecting several plants, including tomatoes. Necrotic blemishes and twisted and fern-like stems define ToMV infection (Broadbent, 1976; Xu et al., 2021).

## Methodology

**Figure 3:**
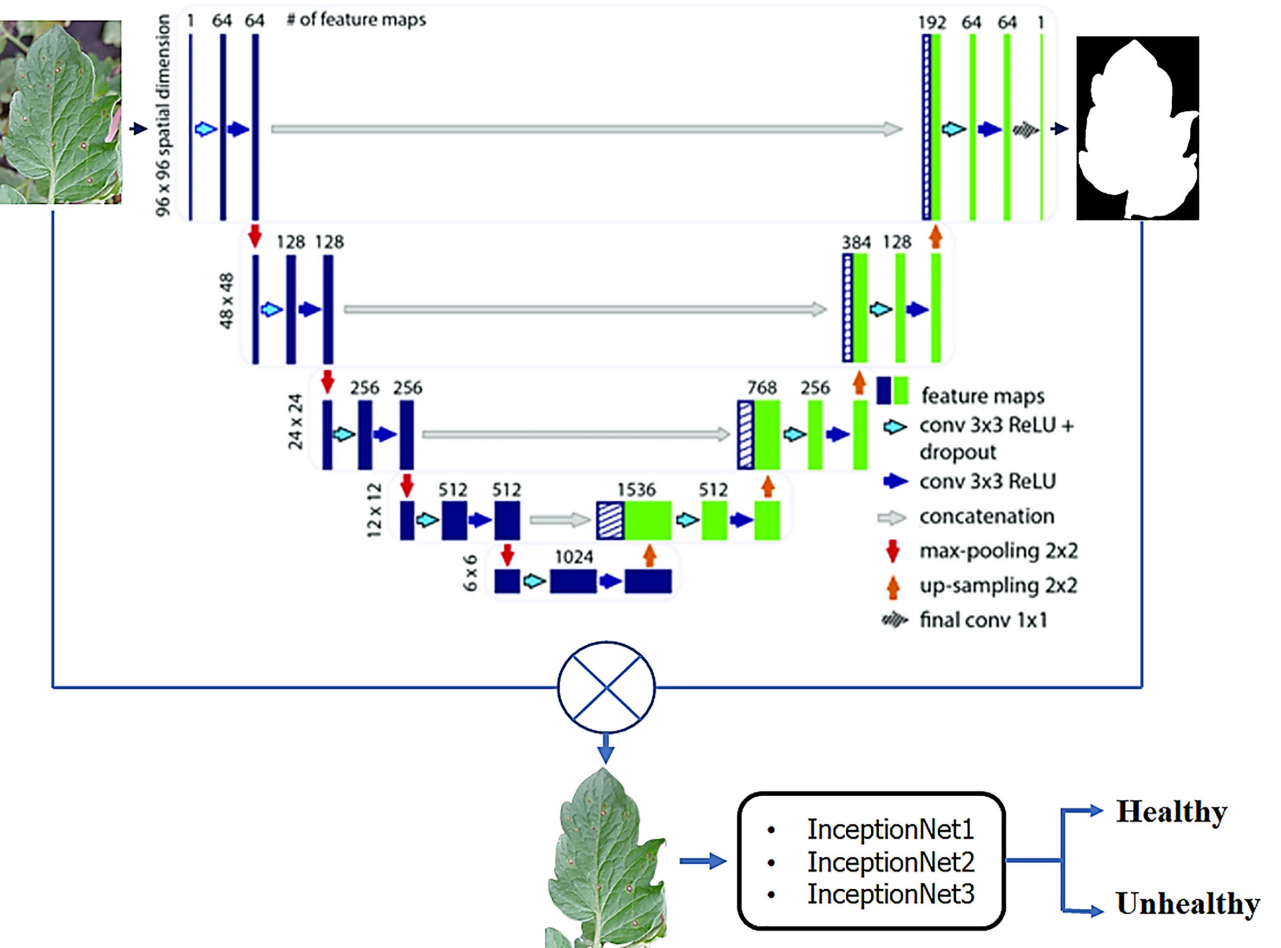
Proposed Tomato Plants leaf diseases Classification Model.

The proposed framework is summarized in the given below **Figure 3**. The dataset used in this research-based project comes from the village of plant benchmark dataset (Hughes and Salathe, 2015; SpMohanty, 2018); the dataset consists of the leaf and their segmented mask images. As discussed in the above sections, this work is performed using three different classification strategies i.e.

(1) the binary classification that only classifies the leaves into healthy and non-healthy classes.
(2) the experiment was performed on five unhealthy and one class of healthy segmented images of leaves.

The paper also investigates the most effective segmentation network for background leaf segmentation is the U-net segmentation model. The segmented tomato leaf pictures are then utilized to verify the cam imagining, which has been proved trustworthy in several applications.

### Overview of the dataset

The proposed models are evaluated using the Plant Villag dataset consisting of 18161 images and segmented binary mask images (Hughes and Salathe, 2015). The Plant Village is benchmark and widely used dataset utilized for the training and validation of the classification and segmentation model.

**Figure 4:**
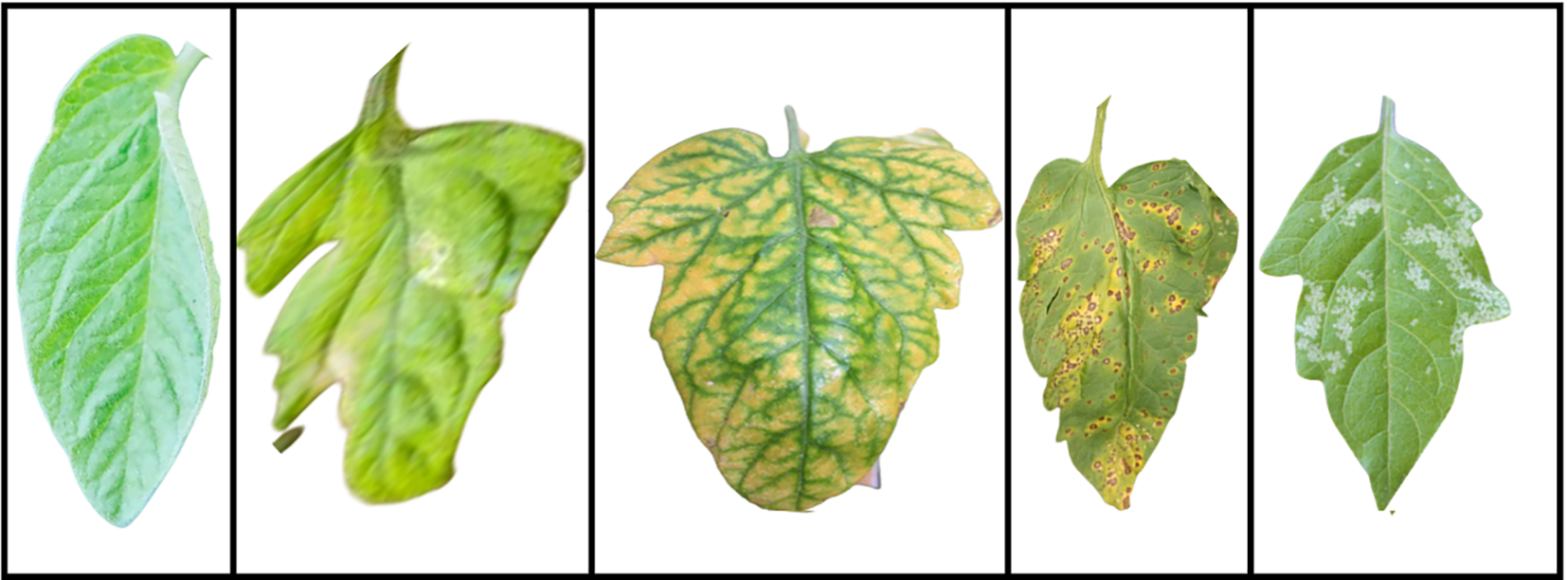
Some random samples of tomatoes leaf images from the benchmar Plant Village Dataset.

This dataset was additionally used to prepare a division and order model for tomato leaves. The images from the dataset were divided into ten classifications where only one class is healthy while all others are from the unhealthy class. The entirety of the pictures was separated into ten classes, one of which was strong and the other nine were damage (e.g., bacterial smear, early leaf mold, leaf shape, leaf mold, and yellow leaf curl infection and the nine undesirable classifications were additionally partitioned into five subgroups (i.e., microscopic organisms, infections, growths, molds, and parasites). **Figure 4** shows some examples of segmented tomato leaf and mask leaf pictures for the healthy and unhealthy classes. **Table 2** additionally incorporates a point-by-point depiction of the number of images in the dataset, which is valuable for the arrangement work debated in the more prominent aspect of the accompanying area.

**Table 2:** Total amount of healty and disease affected tomato leaves images in the Plant Village Dataset.

Types	Normal	Bacteri	Mold	Virus	Fungal	Mite
Classes	Healty(1589)	Bacterial Patches (2131)	Intense Mold (1922)	Curling and Crisping Yellow (5362)	Fungal Pathogens (998)	Tetranychus urticae Koch(1681)
					Septoria lycopersici (1769)	
				Pathogenic virus (Mosaic) (381)	Corynespora cassiicola(1399)	
					Crushed Dry Leaf (Mould) (949)	

### Dataset image preprocessing

#### Image normalization and rescaling

The image input size for various CNN architectures for segmentation and classification Varies. All the images from the dataset for training a U-net model were resized to 256x256x3, while for InceptionNet (1, 2, and 3), the images were resized 299x299x3. CNN networks have input picture size necessities that should be met. All the images in the dataset were normalized using the z-score normalization, where the value of z-score was computed from the standard deviation (SD) and mean of the training images dataset.

#### Image augmentation

As the dataset is imbalanced and doesn’t have equivalent images in various classes, training with an unequal dataset may lead to models’ overfitting or underfitting issues. The number of pictures in all the classes is kept equal by augmenting (increasing the quantity of image data) the images. An equal number of images in all the programs (balance dataset) can train a reliable model to provide better performance accuracy (M. H. Chowdhury et al., 2019; Chowdhury et al., 2020; Rahman et al., 2020; Rahman et al., 2020; Tahir et al., 2022).

Three types of augmentation are applied to the image data, i.e., image rotation, image translation, and image scaling, to create a balanced dataset using data augmentation. To apply rotation to the training images, the images were rotated in a clockwise and anti-clockwise direction with an angle from 5 to 15 degrees. The scaling of images is zooming in or zooming out of an image; in our case, the scaling up and scaling down percentage is 2.5 to 10. The translation of an image is the processing of changing the location of objects in an image; the leaf region is translated horizontally and vertically by a percentage of 5-15.

### Experiments

#### Leaf segmentation

To determine and select the best leaf segmentation model, various U-net segmentation models are trained. K-Fold cross-validation method is applied to split up the data into trainset and a test set. The value of K is 5, which means that each model will be trained five times and validated five times; in each fold, 80% of the data (leaf and segmented mask images) will be used for training the model while the remaining will be used for model validation. (**Table 3**).

**Table 3:** Details of benchmark dataset used for proposed model performance evaluation.

Dataset name	No. of image and their ground truth mask of tomato leaves	Size of training set	Size of validation set	Size of testing set
Plant Village tomato leaf images	18159	13082	1447	3628

The trainset and test set class distribution are equal. As we know that each fold consists of 80% of the data for model training. So out of 80%, 90% of the data is going for model training, while the remaining 10% will be used for model validation which will assist the model in avoiding the overfitting issue. In this research, three state-of-the-art loss functions, i.e., Binary Cross-Entropy Mean-Squared Error loss and Negative Log-Likelihood, are performed to choose the optimal act evaluation metrics used to select the best segmentation model for tomato leaves.

Moreover, a proposed model training stunting condition is reported in some updated research work. If there is no improvement in the validation loss for the first five epochs, the model training should be immediately stopped (Chowdhury et al., 2020; Rahman et al., 2020; Tahir et al., 2022).

#### Classification of tomato leaf diseases

This research study explored a deep learning-based system that used a newly built convolutional neural net called Inception Net to categorize segmented tomato leaf disease pictures to improve disease detection accuracy. Three distinct types of picture categorization tests were conducted as part of this study. As shown in **Table 4**, the images utilized in the analyses for training multiple classification models using the segmented leaf images were taken from different sources. **Table 5** contains a summary of the experiment’s parameters, as well as the results of the study into picture classification and segmentation techniques.

**Table 4:** Quantitative analysis of proposed classifiers experimental work.

Classification	Types	No of images	Segmented and non-segmented images		
			Training images	Validation images	Testing images
Binary Class	Healthy	1519	1095x10 = 10950	121	303
	Affected	16750	12075	1340	3350
Multi-Class(6 Classes)	Healthy	1519	1093	121	303
	Fungi	5115	3682	409	1023
	Mold	1898	1366	151	379
	Virus	5744	4135	459	1148
	Bacteria	2154	1550	172	430
	Mite	1839	1324	147	367
Different classes(Ten classes)	Healthy	1519	1093	121	303
	Early Blight	1050	756	84	210
	Target spot	1454	1046	116	290
	Septoria leaf spot	1721	1239	137	344
	Bacterial spot	2177	1567	174	435
	Leaf Mold	902	649	72	180
	Late Bright Mold	1960	1411	156	392
	Tomato yallow leaf curl virus	5307	3821	424	1061
	tomato mosic virus	373	268	29	74

**Table 5:** List of Hypermaters, loss and optimizer used for training classification and segmentation models.

Parameters	Segmentation model	Classification model
Batch size	32	64
Learning Rate (Initial)	0.0001	0.0001
Epochs	45	50
Shuffle Each Iteration	Yes	Yes
Stopping criteria	5	10
Loss function	Negative Log Likelihood Loss/MSE	CELoss
Optimizer	SGDM	SGDM

#### Inception-V1.

InceptionNet is a collection of deep neural networks that were developed using the Inception module. The initial edition of this series, Googlenett, is a 22-layer deep network. The Inception module is built on the idea that neurons with a shared objective (such as feature extraction) should learn together. In the bulk of early iterations of convolutional architecture, the main focus was on adjusting the size of the kernel to obtain the most relevant features. In contrast, InceptionNet’s architecture emphasises parallel processing as well as the simultaneous extraction of a number of different feature maps. This is the trait that most distinguishes InceptionNet from all other picture categorization models currently available.

#### Inception-(V2 and V3)

Inception v2 and Inception v3 are presented in the same paper. There exists an initial architecture inception-V1 where inception-V-2 and V-3 are widely used in the literature as transfer learning methods to solve various problems. For the Inception part of the network, we have 3 standard modules of 35×35 filter size, each with 288 filters in a layer. This is reduced to a 17 × 17 grid with 768 filters using a grid reduction technique. In inception-V3, the decomposed 5 initial modules, as shown, are reduced to an 8 × 8 × 1280 grid using the grid reduction technique. A grid reduction technique is used to reduce this to an 8 × 8 × 1280 grid. The inception-V3 model consists of two coarsest 8 × 8 levels of the Inception module, and each block has a tandem output filter bank size of 2048. The detailed architecture of the network, including the size of the filter banks in the Inception module, is given in the base research paper (Szegedy et al., 2014).

The intuition is that the neural network performs better when the convolution does not significantly change the input dimensionality. Too much dimensionality reduction may lead to information loss, called a “representation bottleneck”. Using intelligent decomposition methods, convolution can be made more efficient in terms of computational complexity. To increase the computational speed, the 5x5 convolution is decomposed into two 3x3 convolution operations. Although this may seem counterintuitive, the cost of a 5x5 convolution is 2.78 times that of a 3x3 convolution. Therefore, stacking two 3x3 convolutions can improve performance.

This translation was created with the assistance of the DeepL.com Translator (free version)

All of the experiments were performed on an Intel-based corei7 9th generation CPU with a RAM of 64 GB and NVIDIA RTX 2080Ti 11GB GDDR6 GPU using the python 3.7 popular deep learning framework the PyTorch library.

Performance matrix: Segmentation of tomato leaves: The proposed lesion segmentation model performance evaluation metrics are listed below (2)– (4).


2 $$Accuracy=\;\frac{tp+tn}{tp+fp+fn+tn}$$

3 $$IoU=\;\frac{tp}{tp+fn+fp}$$

4 $$Dice\;Cofficent\;=\;\frac{\left(2*tp\right)}{\left(2*tp+fn+fp\right)}$$

Classification of segmented tomato leaves: The leaf classification performance evaluation metrics are listed below (5)– (9).


5 $$Accuracy=\;\frac{tp+tn}{tp+fp+fn+tn}$$

6 $$Sensitivity=\;\frac{tp}{tp+fn}$$

7 $$Specificity=\;\frac{tn}{tn+fp}$$

8 $$f1-Score=\;\frac{\left(2*tp\right)}{\left(2*tp+fn+fp\right)}$$

In the False Positive and False Negative measurements, you can see the photos of healthy and sick tomato leaves mistakenly identified. The True positive rate (TPR) indicates the number of adequately detected healthy leaf pictures. Although the True negative rate (TN) denotes the number of properly identified diseased leaf pictures, the actual percentage of healthy leaves represented is the Healthy Leaf Volume Index (HVI). Additionally, image segmentation and classification models are compared using Equation No. 9, which depicts the time required to test a single picture.


9 $$T=t^{\prime \prime }-t'$$

Where to denotes when a segmentation or classification model starts to process the image I while t’’ denotes the completion time when an image I am segmented or classified.

## Results

This section details the performance evaluation of different neural network architectures (Segmentation & Classification) in various experiments.

### Tomato leaf segmentation

To segment the tomato leaf pictures, two different deep learning-based segmentation models are employed. Namely, The U-net (Navab et al., 2015) and Modified U-net (Navab, 2020) are two neural networks that are trained and verified using pictures of tomato leaves.

**Table 6** illustrates the presentation of two advanced segmentation deep learning designs tested against one another using various loss functions to demonstrate how effectively they compete against one another (NLL, MSE, and BCE). Notably, the Improved U-net with NLL loss function may have surpassed the unique U-net in terms of the quantity and quality of segments created for the ROI (leaf region) across all images instead of the original U-net.

**Table 6:** Quantitative results analysis of U-Net and Modified U-Net over benchmark dataset.

Loss function	Network	Validation loss	Validation accuracy	Intersection Over Union	Dice	Inference time
NLog	Original U-net	0.0177	96.33	95.43	96.51	13.10
CELoss	Original U-net	0.0167	96.49	95.89	96.42	12.65
Mean Square	Original U-net	0.0135	96.62	96.33	97.76	12.41
NLog	Improved U-net	0.0067	98.88	98.65	98.91	11.20
CELoss	Improved U-net	0.015	97.91	97.77	96.93	11.01
Mean Square	Improved U-net	0.069	98.21	98.12	98.54	10.98

**Figure 5:**
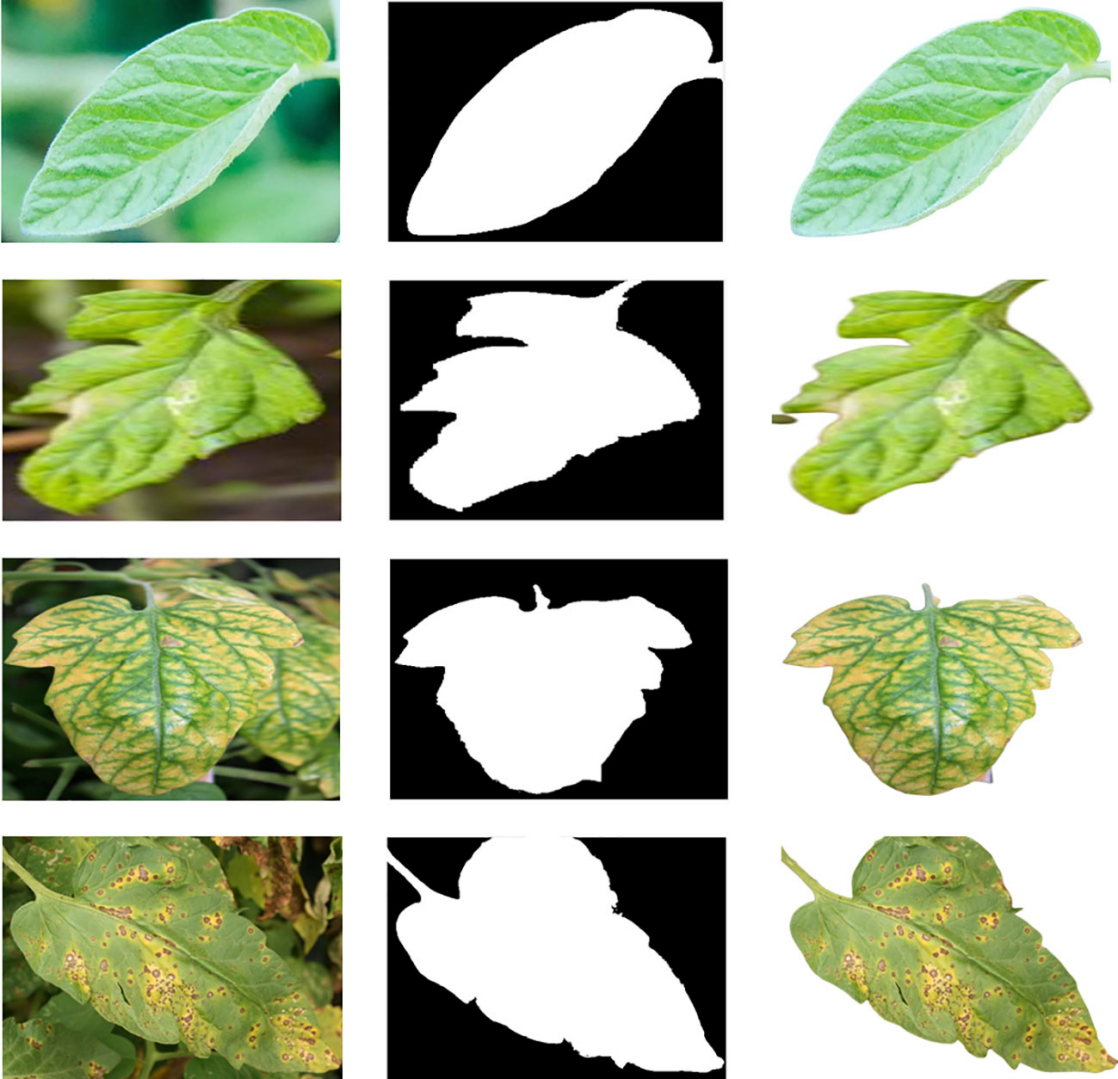
Original Tomota Leaf Images, Ground Truth Mask and Segmented Leaf using Modified U-Net CNN Model.

**Figure 6:**
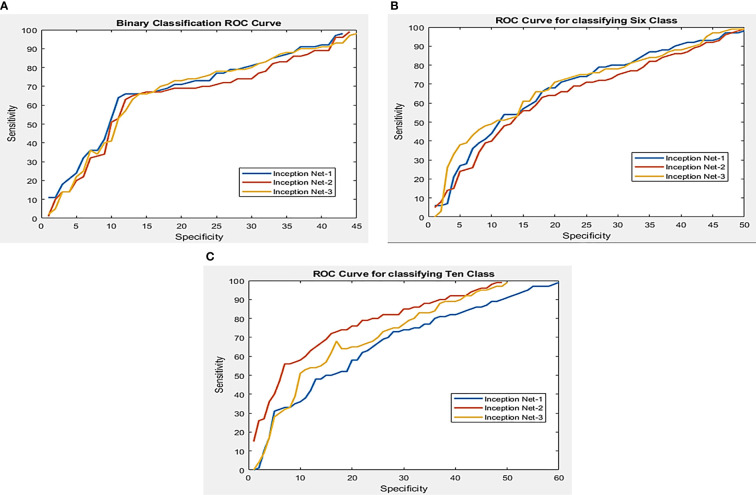
Working feature curves for **(A)** binary segmented leaf classification, **(B)** sixth class segmented leaf classification, and **(C)** tenth class classification of the segmented leaf.

A modified U-net design with a negative log-likelihood loss function was used to segment leaves into leaf regions. The following parameters were computed: validation loss, validation accuracy, IoU, and dice. The results for the Modified U-net model with Negative Log-Likelihood loss function were 0.0076, 98.66, 98.5, and 98.73 for the four variables. Example test leaf images from the Plant Village dataset are shown in **Figure 5** with their ground truth masks. Segmented ROI images were produced using the Modified U-net model with a Negative Log-Likelihood loss function, which was trained on the Plant Village dataset and is displayed in **Figure 6**.

### Classification of tomota plant disease

**Figure 7:**
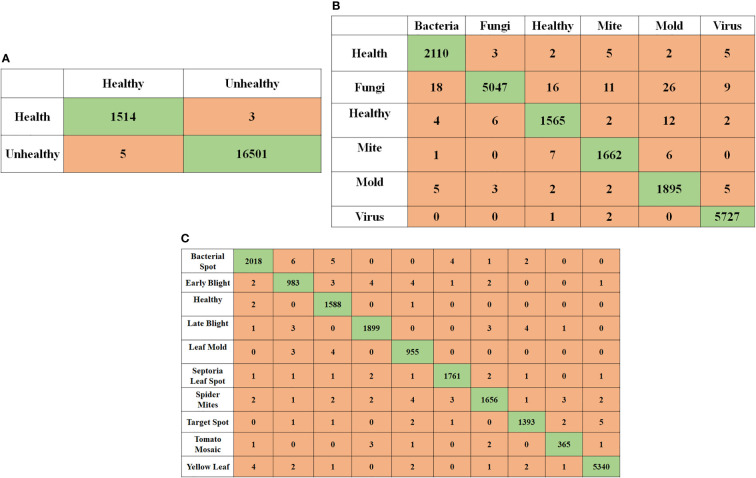
Image classification using compound scaling CNN-based models of healthy and diseased tomato leaves for segmented leaf images **(A)** for 2 class classification, **(B)** for 6 class classification, and **(C)** for 10 class classification.

In this work, three separate tests were done using pictures of segmented tomato leaves, each with a different outcome. Using three distinct Inception Net families, the performance of segmented leaf pictures classified using three other classification techniques such as InceptionNet1, InceptionNet2, and InceptionNet3 is compared in **Table 7**. Pre-trained models perform exceptionally well in identifying healthy and diseased tomato leaf pictures, as shown in **Figure 7**, in problems with two classes, six classes, and ten classes, respectively. In addition, when non-segmented pictures were used, the results were superior.

**Table 7:** Performance analysis of Modified U-Net with InceptionNet CNN models.

No. Classes	CNN Model	Performance Analysis on 90 CI					
		Overall Average					
		accuracy	Precision	Sensitivity	F1 score	Specificity	Inference time
Class 2	InceptionNet-1	99.74 ± 0.03	99.79 ± 0.03	99.71 ± 0.06	99.77 ± 0.05	99.77 ± 0.06	22.19
	InceptionNet-2	99.92 ± 0.01	99.94 ± 0.01	99.92 ± 0.01	99.92 ± 0.08	98.76 ± 0.4	29.71
	InceptionNet-3	99.87 ± 0.08	99.91 ± 0.08	99.87 ± 0.04	99.89 ± 0.03	99. 81± 0.05	39.45
Class 6	InceptionNet-1	97.12 ± 0.9	97.18 ± 0.9	97.12 ± 0.9	97.14 ± 0.23	99.51 ± 0.9	25.77
	InceptionNet-2	98.76 ± 0.18	98.79± 0.5	98.75 ± 0.3	98.76 ± 0.18	99.69 ± 0.06	42.05
	InceptionNet-3	99.32 ± 0.14	99.4 ± 0.19	99.28 ± 0.16	99.35 ± 0.26	99.83 ± 0.08	52.66
Class 10	InceptionNet-1	99.61 ± 0.06	98.72 ± 0.6	98.70 ± 0.8	98.70 ± 0.3	99.85 ± 0.07	44.54
	InceptionNet-2	99.90 ± 0.4	99.91 ± 0.3	99.89 ± 04.	99.90 ± 0.30	99.96 ± 0.08	53.63
	3	99.79 ± 0.08	99.23 ± 0.33	99.65 ± 0.14	99.44 ± 0.21	99.89 ± 0.05	59.85

Aside from ten-class problems, where InceptionNet3 performed marginally better than InceptionNet1 compared to other training models, InceptionNet1 outperformed other trained models when utilizing leaf pictures segmented of two, six, and ten-class issues and without it. We conducted extensive testing on several versions of Inception Net. They observed that when the depth, breadth, and resolution of the network are raised, the performance of the network increases. Because the depth, breadth, and resolution of the network are scaled as the Inception Net model grows in depth, width, and resolution, the testing time (T) grows. In contrast, when the classification scheme grows more complex, the performance of Inception Net’s scaled version does not appear to increase substantially.

A two-class and a six-class problem with InceptionNet1 outperforms the competition in segmenting tomato leaf images, achieving a 99.95 percent accuracy, 99.95 percent specificity, and 99.77 percent specificity for two-class 99.12 percent, 99.11 percent, and 99.81 percent specificity for three-class problems. For its part, InceptionNet3 achieved the highest accuracy, sensitivity, and specificity scores in the ten-class test, with 99.999% accuracy, 99.44 percent sensitivity, and specificity scores in the ten-class test, respectively. **Figure 5** illustrates that increasing the number of parameters in a network result in marginally better performance for 2, 6, and 10 class issues. On the other hand, deep networks can give a more significant performance gain for problems with two and six classes, respectively. **Figure 6** depicts the Images of segmented tomato leaves used to create Receiver Operating Characteristic (ROC) curves for problems involving two-class, six-class, and ten-class difficulties. This section shows how the receiver working features curves for second class, sixth class, and tenth class issues utilizing segmented tomato leaf pictures look.

The Design with NLL loss function produced masks (2^nd^ left), segmented leaf with matching segmentation (2^nd^ right), and ground truth images of tomato leaves are shown in **Figure 5**. (right).

To categorize tomato leaf diseases, segmented and original leaf pictures to get the findings shown in **Table 7**. Italicized outcomes denote the most favorable outcomes).

For the best performing networks depicts the confusion matrix when applying tomato leaf pictures to different classification tasks. Of the six out of 16,570 unhealthy tomato leaf pictures that were correctly classified as healthy, the network with the most outstanding performance, InceptionNet1, accurately categorized them. however, the network with the worst performance, InceptionNet1, incorrectly classified 1591 of them as unhealthy.

Six different classes of unhealthy tomato leaf images, which consisted of one healthy class and five distinct unhealthy classes, were identified in the six-class issue. While only three misclassified images were found in the six-class issue, 1591 healthy tomato leaf images were found in the healthy tomato leaf category. Only one tomato leaf was misclassified in the six-class problem, which consisted of healthy and unhealthy classes of one hundred and ninety-nine different types. There were 16,570 images of diseased tomato leaves to choose from in the six-class assignment. A study discovered that the best network for the ten-class problem was InceptionNet3, which had only four misclassifications of healthy imageries and 105 misclassifications of unhealthy images in healthy tomato leaf images.

### Cam-Score ROI visualization

**Figure 8:**
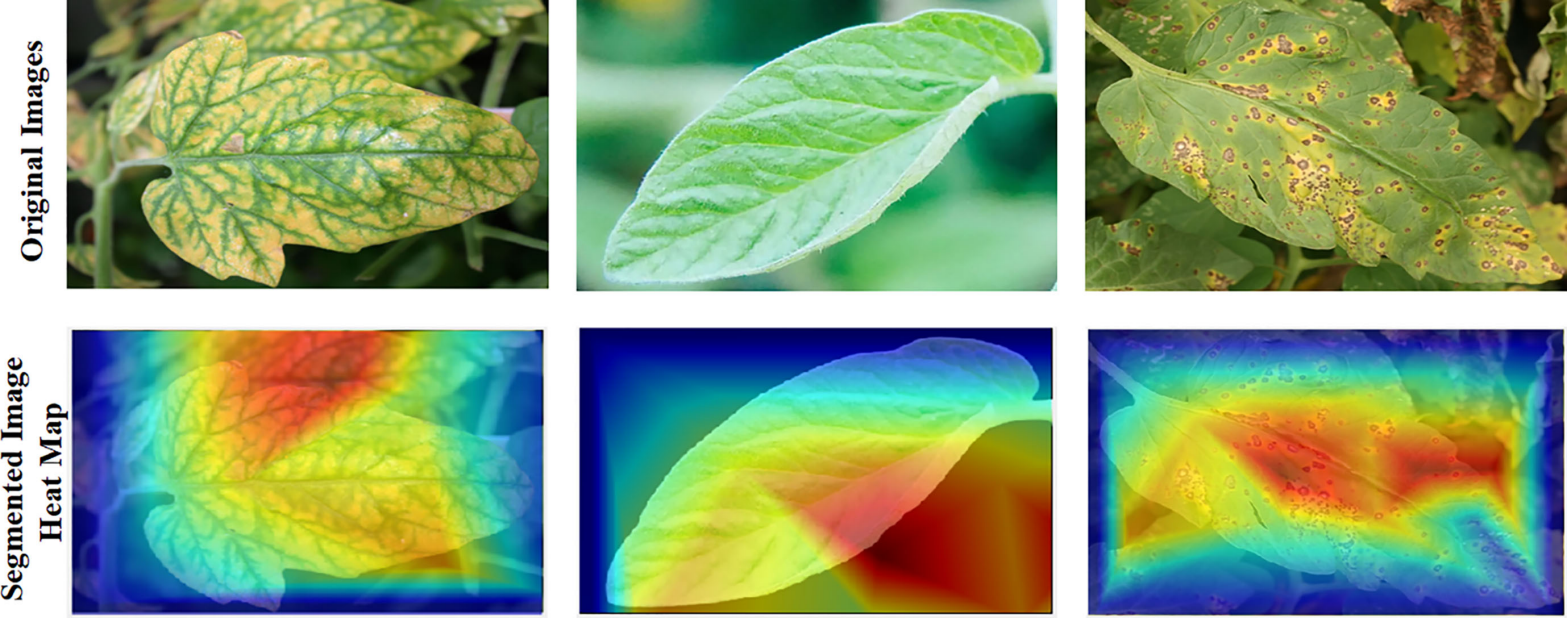
Accurate classification and visualization of ROI using the CAM-Score tool: The red intensity indicates the severity of the lesion.

**Figure 9:**
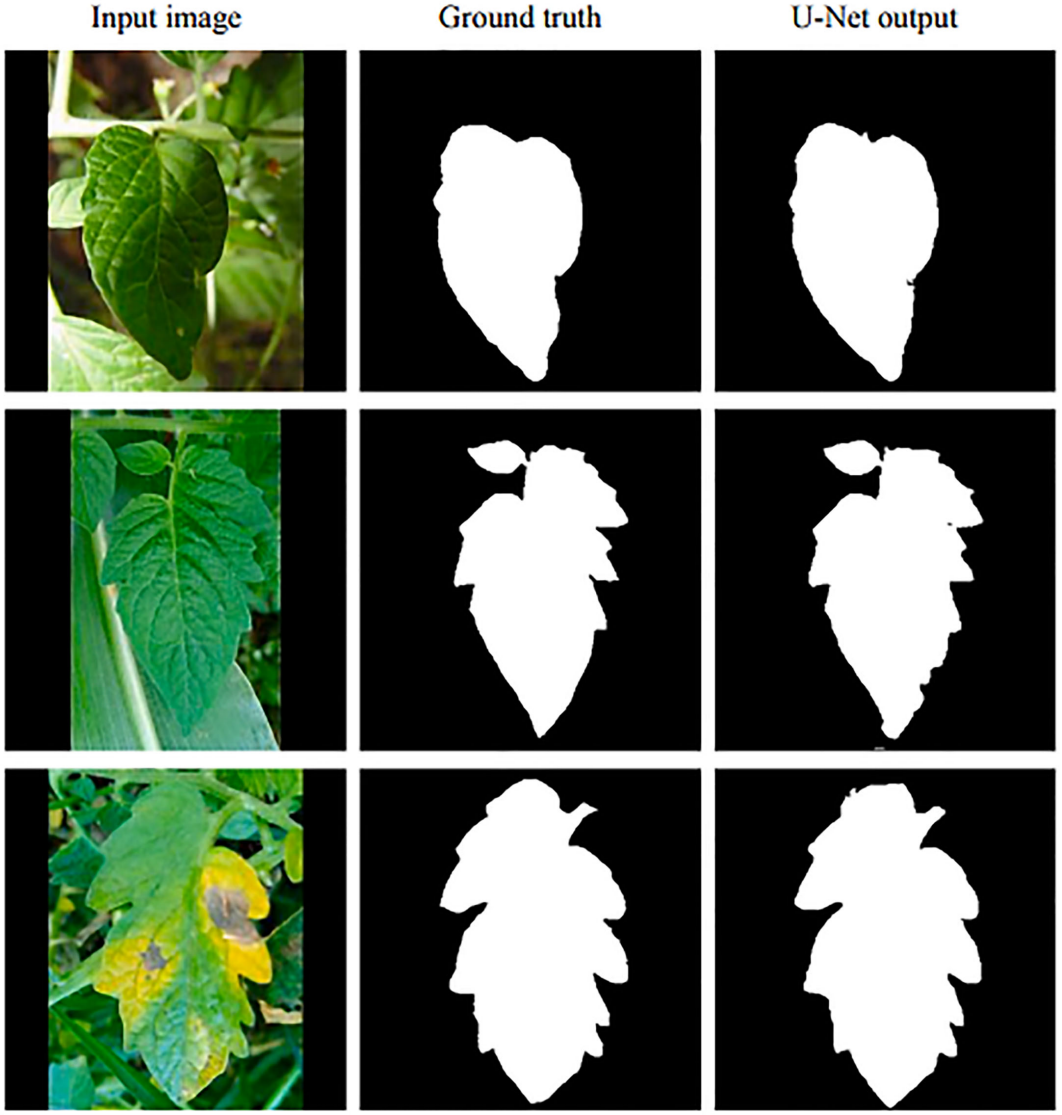
Visual Results of Proposed Modified U-Net CNN model.

**Figure 10:**
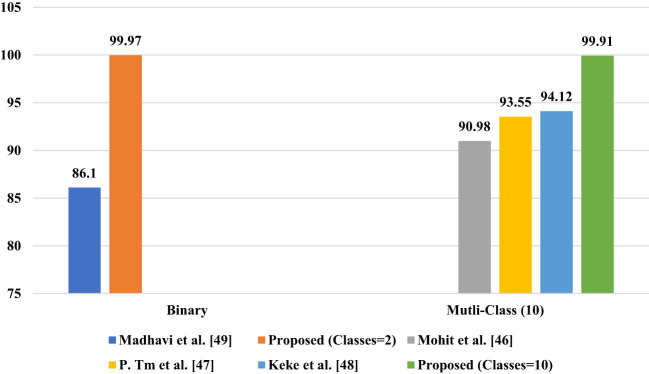
Proposed Model Comparison with state-of-the-artwork.

The dependability of trained networks was evaluated in this study through visualization tools based on five distinct score-CAM categories; it was determined that they were either healthy or sick. The 10-class problem was solved using temperature maps created from tomato leaf segmented pictures. **Figure 8** depicts the unique tomato leaf samples and the temperature maps created on segmented tomato leaf segments. **Figure 8** illustrates how the networks learn from the leaf pictures in the segmented leaf, which increases the reliability of the network’s decisions. Doing so contributes to disproving the notion that CNN makes choices based on irrelevant variables and is untrustworthy (Schlemper et al., 2019). **Figure 9** further illustrates how segmentation has assisted in categorization, with the network learning from the area of attention resulting from segmentation. Using this reliable learning method, we could classify erroneous information correctly. Comparing segmented pictures to non-segmented images, we found that division assisted in knowledge and making judgments from germane areas associated with non-segmented imageries see **Figure 10**.

## Discussion

Plant diseases pose a substantial danger to the global food supply. The agricultural industry requires cutting-edge technology for disease control, which is currently unavailable. The application of technologies based on artificial intelligence to the identification of plant diseases is currently the subject of intensive research. Popularity of computer vision-based disease detection systems can be attributed to their durability, ease of data collection, and quick turnaround time. In this study, classification and segmentation of tomato leaf images are used to evaluate the performance of model scaling CNN-based architectures relative to their predecessors. The initial categorization (Healthy and Unhealthy) employed a two-category classification; subsequently, a six-category classification was employed (Healthy, Fungi Bacteria Mould Virus, and Mite). Prior to the completion of the final classification, a preliminary two-class classification (Healthy and Unhealthy) was utilized (Healthy, Early blight, Septoria leaf spot, Target spot, Leaf mold, Bacterial spot, Late bright mold, Tomato Yellow Leaf Curl Virus, Tomato Mosaic Virus, and Two-spotted spider mite). This study determined that the InceptionNet1 model was the most successful across all classes, outperforming all others with the exception of binary and segmented image classification, which the InceptionNet1 model outperformed. Utilizing segmented photographs and binary classification, this model outperformed all others in binary classification and 6-class classification using segmented images. This model performed significantly better than other models at classifying segmented 6-class images.

InceptionNet1 has an overall accuracy of 99.5% when using segmented images to classify sick and healthy tomato leaves into two classes. Using a 6-class classification method, the InceptionNet1 algorithm achieves an overall accuracy of 99.12%, according to the study’s findings. InceptionNet3 demonstrated an overall accuracy of 99.89 percent on a 10-class classification test involving segmented images and photos. **Table 8** highlights the article’s findings, which are comparable to the current state of knowledge in their respective fields of study. The Plant Village dataset utilized in this study consists of images captured in a variety of environments; however, it was collected in a specific location and only contained images of specific tomato varieties. Using a dataset consisting of images of tomato plant varieties from around the world, a study was conducted to develop a more robust framework for identifying early illness in tomato plants. In addition, due to their simpler design, CNN models may be useful for testing portable solutions with non-linearity in the removal layer feature.

**Table 8:** Proposed model performance comparsion with other state-of-the-art work.

Article	Classification	Dataset	Accuracy	Precession	Recall	F1 score	Result
(Agarwal et al., 2020)	Multi-Class (10)	Plant village	90.98%	89%	91.97%	90.96%	Non segmented
(G Irmak and Saygili, 2020)	Multi-Class (10)	Plant village	93.55%	93.93%	95.69	93.91%	Segmented
(Zhang et al., 2018)	Multi-Class (10)	Custom	94.12%	94.72%	94.35%	96.64%	Non segmented
(Dookie et al., 2021)	Binary	Custom	86.10%	86.44%	86.37	86.41%	Non segmented
Proposed study	Binary	Plant village	99.97%	99.11%	99.96%	99.93%	Segmented
	Multi-Class (6)	Plant village	99.22%	99.19%	99.20%	99.17%	Segmented
	Multi-Class (10)	Plant village	99.91%	99.31%	99.29%	99.30%	Segmented

## Conclusions

This study presents the outcomes of a CNN built on the recently proposed Inception Net CNN architecture. The CNN model was effective, and which accurately assign a class label to a tomato leaf image as healthy or non-healthy. The reported results were obtained using the benchmark publicly available Plant Village dataset (Hughes and Salathe, 2015), demonstrating that our model outperforms a number of current deep learning techniques. Compared to other architectures, modified U-net was superior at separating leaf images from the background. In addition, InceptionNet1 was superior to other designs in removing high-priority features from snaps.

In addition, when the systems were trained with a greater number of parameters, their overall performance significantly improved. Using trained models may allow for the automated and early detection of plant diseases. Professionals require years of training and experience to diagnose an illness through a visual examination, but anyone can utilize our methodology, regardless of their level of experience or expertise. If there are any new users, the network will operate in the background, receiving input from the visual camera and immediately notifying them of the result so they can take the appropriate action. As a result, preventative measures may be taken sooner rather than later. Utilizing new technologies such as intelligent drone cameras, advanced mobile phones, and robotics, this research could aid in the early and automated detection of diseases in tomato crops. By combining the proposed framework with a feedback system that provides beneficial recommendations, cures, control measurement, and disease management, it is possible to increase crop yields. Work will be expanded to evaluate the performance of the proposed method in an embedded system and camera-based real-time application. The real-time system will be a hardware product that, after training with deep learning, will monitor and predict the health of plants.

## Data availability statement

The original contributions presented in the study are included in the article/supplementary material. Further inquiries can be directed to the corresponding authors.

## Authors contributions

MS conceptualized of this study, conducted experiments,wrote the original draft, and revised the manuscript. TH wrote the manuscript and performed the experiments. BS made the experimental plan, supervised the work and revised the manuscript. IU performed the data analysis and reevised the manuscript. SS made the experimental plan and revised the manuscript. FA evaluated the developed technique and revised the manuscript. SP designed the experimental plan, supervised the work andrevised the manuscript. All authors have read and agreed to the published version of the manuscript.

## Funding

This work was supported by the DGIST R&D program of the Ministry of Science and ICT of KOREA (22-KUJoint-02, 19-RT-01, and 21-DPIC-08), and the National Research Foundation of Korea (NRF) grant funded by the Korean Government (MSIT) (No. 2019R1C1C1008727). This research work was also supported by the Cluster grant R20143 of Zayed University, UAE.

## Acknowledgments

We thank all the authors for their contribution.

## Conflict of interest

The authors declare that the research was conducted in the absence of any commercial or financial relationships that could be construed as a potential conflict of interest.

## Publisher’s note

All claims expressed in this article are solely those of the authors and do not necessarily represent those of their affiliated organizations, or those of the publisher, the editors and the reviewers. Any product that may be evaluated in this article, or claim that may be made by its manufacturer, is not guaranteed or endorsed by the publisher.
